# The Contribution of Long-Term Mindfulness Training on Personal and Professional Coping for Teachers Living in a Conflict Zone: A Qualitative Perspective

**DOI:** 10.3390/ijerph17114096

**Published:** 2020-06-08

**Authors:** Tal Litvak-Hirsch, Alon Lazar

**Affiliations:** 1 Conflict Management & Resolution Program, Department of Multidisciplinary Studies, Ben-Gurion University of the Negev, Beer Sheva 8410501, Israel; 2 Program for Education, Society and Culture, Ono Academic College, Kiryat Ono 5545173, Israel; alon.la@ono.ac.il

**Keywords:** coping, mindfulness, teachers, terror

## Abstract

It has been suggested that mindfulness training can provide teachers with coping mechanisms and influence their perceptions of self and others. However, how does mindfulness help teachers cope in a stressful security situation both as Israeli citizens who live in a war zone and as teachers who are responsible for their students’ lives? Fifteen female teachers, who lived and worked in the western Negev and who had completed two-years of mindfulness training, were interviewed. Interviewees reported that their coping skills had been heightened as result of being able to put aside intrusive thoughts and feelings that used to paralyze them and to focus on active coping, centered on what they needed to do promptly. Most also noted a more accepting attitude of themselves, without self-criticism or blame for what they should have or should not have done when facing the stressful situation. In relation to their students, they were more accepting of the behaviors and emotions expressed by their students and reported being more compassionate. The results will be discussed through the prism proposed by Lazarus and Folkman (1991). Educational implications of the outcomes of mindfulness training for those living in areas under the shadow of war will be suggested.

## 1. Introduction

The past few decades have witnessed a growing interest in research on both the negative and the positive outcomes of ongoing exposure to terror and war, particularly with regard to the coping mechanisms of both the population at large and professionals living in such areas [1,2].

Israeli society, whose members experience such situations, struggles to remain vibrant and flourishing while threatened by war and terror. In recent years, the situation has become particularly acute for those living within a 40-km range from the Gaza Strip, namely, in Israel’s western Negev. These citizens face rocket bombardments and flaming balloons on an ongoing basis. The current study focuses on the reactions of teachers who live and work in this area and have undergone mindfulness training, aiming to assess their coping skills as individuals and professionals. In the literature review, we begin with stress and coping theory. We then discuss mindfulness, and finally, focus on ways of coping and mindfulness among teachers.

### 1.1. Stress and Coping

In the transactional model of coping [3], stress is conceptualized as a situation in which a person feels that s/he is forced to garner considerable resources in order to face external and/or internal demands.

The model also suggests that when attempting to cope with a stressful situation, two cognitive appraisals, primary and secondary, are employed. Primary appraisal is directed by the question, “What do I have at stake in this encounter?”, and is accompanied by emotions such as fear, worry, anger, and shame. The question, “What can I do or what are my options for coping?” marks secondary appraisal. During these appraisals, problem-focused and emotion-focused coping are available to the individual. Problem-focused coping means that the individual supposes that the situation can be dealt with effectively, while emotion focused coping directs the individual to assign new meanings to the stressful situation and restrain and handle negative emotions [3].

### 1.2. Mindfulness and Coping

A key element of mindfulness training is the cultivation of skills for dealing with challenging, uncertain, and stressful situations, by bringing the individual to focus their attention to a certain purpose, as it takes place in the present moment, and acting non-judgmentally toward the experience [4]. This is made possible as a result of the non-judgmental curiosity encouraged by mindfulness, which promotes attention to the stream of consciousness without emotional reactivity, mental rigidity, or rejection [5].

In accord with the transactional model of coping [3], it has been suggested that mindfulness enhances clarity and accuracy in the assessment of both the stressor (primary appraisal) and the available resources (secondary appraisal), resulting in more effective coping responses [6]. Mindfulness often forms a connection with compassion.

Compassion denotes focusing one’s attention on the other, aiming to assist, and it is based upon positive feelings [7]. Findings suggest that short-term compassion training increased positive affect toward a suffering other [8].

Mindfulness is regarded as one of the keys to self-compassion, since mindfulness aids people to become aware that they are struggling and encourages them to act with kindness toward themselves [9]. Furthermore, when individuals treat themselves with self-compassion, they have more to give to others, and the loving connected presence that they feel for themselves will resonate on others [10]. Self-compassion may be a valuable coping resource for people experiencing negative life events [11]. It relates most strongly to positive cognitive restructuring and involves thinking about stressful situations in ways that enhance coping [12]. This suggests that practicing mindfulness can encourage compassion toward the self as well as toward others.

The effects of mindfulness training have been extensively discussed in areas such as health and education, noting its positive outcomes with regard to stress reduction [13], enhanced self-compassion, and a shared sense of greater self-awareness and self-acceptance, all leading to an improved capacity for engaging with the present in ways that reduce critical self-judgment [14].

### 1.3. Teachers and Mindfulness Training

The teaching profession is noted for its’ complex nature [15]. Teachers are required to meet a wide range of demands and responsibilities that require skillful social and emotional conduct such as providing emotionally responsive support to students, cultivating a nurturing classroom environment, modeling exemplary emotion regulation, coaching students through conflict situations with sensitivity, successfully (yet respectfully) managing the challenging behaviors of disruptive students, and handling the growing demands imposed by standardized testing. Studies assessing the effects of mindfulness-based interventions for teachers have found consistent improvements in emotion regulation and mindfulness [16], lowering of anxiety [17], more positive handling of job stress, and the tendency to evaluate challenging students in a more positive affective light [18]. In addition, mindfulness training for teachers was found to promote their compassion, self-compassion, and care [19], and to lead to an improvement in their relations with the students and a better classroom climate and management [20].

There is enough evidence attesting to the positive outcomes of participation in mindfulness training when it comes to teachers. However, the existing literature relies upon American samples of teachers, who live in tranquil environments [16,20], rather than their counterparts in conflict zones, who are exposed to terror on a daily basis, as is the case with many teachers in Israel, especially those residing in the western Negev.

### 1.4. Stress and Coping by Israelis Residing in Western Negev, Israel

Beginning in 2008, residents of the western Negev, Israel, have lived under the threat of terror attacks directed to them from the bordering Gaza Strip in the form of missiles, balloon and kite-borne bombs, and more recently bomb carrying drones.

There is a plethora of studies aiming to identify the reactions of Jewish-Israelis living in the area. A review in [21] indicated that area residents exhibit fairly high levels of Post Traumatic Stress Disorder (PTSD) and depression during temporary breaks in the conflict and these levels rise significantly during periods of escalation. In contrast, very little research has studied the coping modes of these residents. One study found that Israeli women living in proximity to the Gaza Strip have been found to use both problem-focused and emotion-focused coping as well as optimism, humor, and denial [1]. In another study, the relations between PTSD and Post Traumatic Growth were found to be mediated by problem-focused coping among the area inhabitants [22].

### 1.5. Mindfulness Training for Teachers in the Western Negev, Israel

To aid teachers in the western Negev, mindfulness training programs have been offered by Sapir College and the Shaar Hanegev Psychological Clinic since 2015. For the first three years, the programs lasted 12 weeks, similar to programs offered to teachers in the USA [23]. These initial stages did not undergo evaluation. In 2018, the program was extended to include a two-year training period in order to provide participants with more tools and techniques. During the first year of the program, between September and June 2018, training was held every second week for three hours. During the intermissions between each meeting, participants were asked to practice and reflect upon the skills they had learned.

For the first two months, the focus was on Buddhist principles and the concepts of compassion and awareness. In the following months, participants practiced various techniques of meditation. These included breathing meditation, in which teachers learned to focus their awareness on their breathing experience; open awareness meditation aiming to bring participants to an awareness and focus upon the here and now, and current feelings, thoughts, and body sensations; and compassion training. Here, each participant was asked to think first of another person, one they felt very close to, and how they approached that person in a kindly manner. Participants were then asked to think about how these warm and kind feelings could be extended and directed toward others including strangers, and in the final stage of the training, how to direct such feelings to those one has difficult relations with.

During the months of July and August, when meetings were not held, participants were asked to continue daily meditation individually, at their homes. In the second year of training, between September and June 2019, each session was held for three hours each second week. These meetings, like the first year of the program, aimed at assisting participants to become more confident in their meditation practices, and to explore further Buddhist principles. In addition, the teachers became familiar with modes of teaching their students the basic principles of mindfulness, mainly, by focusing on the here and now, and discussed their practice with their instructors.

The teachers joined the program on a voluntary basis and received credits from the Israeli Ministry of Education for their attendance, as the course is recognized for its contribution to advanced professional training.

The present study aims to provide preliminary answers to an under-discussed topic in the literature: What is the contribution of a long-term mindfulness program to teachers’ coping as individuals and as teachers living in a conflict zone?

## 2. Materials and Methods

### 2.1. Participants and Procedures

According to the most recent report of the Israeli Ministry of Education, female teachers comprise 85% of the teaching profession in Israel [24]. There are no available public records recording their exact numbers in the western Negev.

All teachers living in the western Negev and eligible to participate in teachers continuing education programs offered by the Israeli Ministry of Education were given the chance to participate in this program. Past research has indicated that Israeli teachers prefer short continuing educational programs that are closely related to their teaching profession [25]. In this respect, the participants in the program discussed in this study are likely to differ from typical participants in the programs offered by the Israeli Ministry of Education. Fifteen female teachers took part in the program and were informed at its beginning that they could leave at the end of the first year. All completed the program and agreed to take part in the research after they were approached by their instructors, who informed them of the study. The teachers received no form of reimbursement for their participation in the study.

The teachers ranged in age between 45 and 57 years old. Of them, four were kindergarten teachers, five were elementary school teachers, and six were high school teachers. Except for two (one divorced, one widowed), all were married, had children, and lived in cities, towns, moshavim, and kibbutzim, at a distance of a few kilometers to 30 km from the Gaza Strip. After receiving the institutional review board IRB approval from the Ben Gurion University Board (2019-06), the teachers were approached by the first author and her graduate students, and were all interviewed in depth, using semi-structured interviews lasting 1–2 h. Interviews were taped and transcribed verbatim.

The interviews were conducted between the end of June 2019 and August 2019, two weeks to a month and a half after the end of the program. Interviews were chosen to assess the participants’ experiences following the program, as qualitative methods such as interviews in mindfulness training assessment have been recommended for their ability to more fully grasp the complexities of a subjective nature as experienced by participants in such programs, that are otherwise not detected by quantitative research [26]. Thus, interviews make it possible to underpin what participants feel, what they have experienced, and how they make sense of the practices they have acquired [27].

The interview guide was designed to explore coping practices employed by the participants and the contribution to these of mindfulness.

Each interview began with the introductory demographic question “Please tell me about yourself”, and this provided information about the participants’ age, family status and number of children, number of years living in the area, number of years as a teacher, and so forth. The teachers were then asked to explain why they decided to join the program, given that it was time consuming and required considerable dedication. The question aimed to identify the profile of the participants in terms of age and teaching experience, and their psychological, emotional, and/or practical expectations regarding the outcomes of the training, considering the fact that the program differs significantly from other programs offered by the Ministry of Education. The third question aimed at learning about the personal experiences of the participants as residents of an area struck by ongoing terror. They were asked to discuss their modes of coping, and emotions and behaviors when encountering difficulties at the personal, family, and/or professional levels. Finally, the teachers were asked to discuss whether, in their opinions, they had detected any impact of their participation in the program on their modes of coping as individuals and/or professionals. In combination, these questions aimed to understand how professionals living in an area struck by terror understand and employ the principles and insights they acquired during their mindfulness training.

### 2.2. Analysis

The thematic approach [28] was applied. This approach suggests looking at each interview as a holistic unit and then tracing the main themes that emerge from the material according to the research questions. After each of the researchers read the interviews and identified the themes for each question, the themes identified independently were compared to assess inter-rater agreement, reaching 0.94 (k = 0.94) and the discrepancies were discussed until reaching agreement [29]. The themes accepted by the research team are presented in the following.

### 2.3. Research Ethics

The teachers were interviewed only after receiving the IRB approval from the Ben Gurion University board (code number 2019-04), and they were informed of the aims of the research, and that they could stop their participation in the interview without the need to explain their decision. All agreed to take part in the study. In order to ensure their anonymity, responses are presented using pseudonyms, with no mention of any identifying information such as age, place of residence, number of children, and so forth.

## 3. Results

### 3.1. Motivation to Join the Training

All of the teachers had heard about mindfulness before their participation in the program, yet none had practiced it, and they had joined the program in order to acquire new skills needed to assist themselves and their students in coping with life in a conflict zone. As Shira suggests, “I have taught science for many years, and I feel confident about my knowledge of the subject. I feel the need to expand my knowledge of skills and abilities required to help my students and myself in stressful situations, as is the case with rockets bombarding our schools and homes”.

### 3.2. Contribution of Mindfulness Training for the Teacher’s Coping Skills

The teachers discussed four life domains that they felt had improved following their participation in the program. First, many of the teachers noted an enhanced acceptance of themselves, free of self-criticism or blame. Sigal explained, “As a result of my mindfulness training, I learned to accept myself, to be less critical of myself. I accept myself and my fears; I am not angry with myself anymore; I do what I can, and it is good enough”.

Second, improved skills needed to cope with stressful events related to their family life were indicated. All noted that when facing a potential heated argument with a family member, they took a moment to relax before acting, and this was attributed to the impact of their participation in meditation sessions. Rachel pointed out, “Following the program, I reminded myself to take a breather, to stop for a moment, to be in the here and now, to avoid an impulsive reaction. I manage to view the stressful situation from a distance”. Miri added: “My husband was an army officer for many years, and as a result, I used to run the home my way, as he was at home only for a few days each month. During his stay, he demanded that things only be done his way. That was a source of ongoing tension. Following the program, I’ve learnt to let go, to breathe and distance myself from the situation, to allow him to make his role at home clear. We became more relaxed and easygoing. He has recently become a civilian, and this transition, and the fact that he is now at home all the time and all that entails, happened smoothly”. However, the teachers noted that these changes did not occur immediately, but rather were a result of their ongoing attendance in the program, as best exemplified by Ronit, “The effects of mindfulness cannot be achieved in one smooth breath; they requires effort, time and practice”.

The third life domain discussed was coping with life in a conflict zone. When relating to the stressful security situation, the interviewees reported that, following their participation in the program, their ability to face their daily stressful reality had improved. Rachel noted that “we have lived in this war zone for years; there are ups and down, with stressful periods and those that are more tranquil. You find your way to manage this situation; yet it is always a challenge. Participation in the program gave a set of skills I was not aware of, and for me, they do the job”.

A skill mentioned as relating to stressful situations was strengthening the awareness of the situation and of the self as part of the situation. Yaara explained, “During the training, I expanded my awareness, first of my breathing. That helped me focus on what was going on inside me in the here and now, looking at the stressful situation from a little distance instead of running into it with full emotional reactions. That was very helpful when the sirens sounded, and you had to focus on action. Regarding the stressful security situation, the interviewees reported that as a result of the intervention, their coping had been aided by the acquired ability to put aside the intrusive thoughts and feelings that served to paralyze them—or at least to ‘soften their volume,’ as Yael explained—and to focus on active coping in the here and now such as running to the shelter, helping the students, and more. Another example of the contribution of mindfulness to active coping was given by Irit: “As a result of the training, I learned to understand that my thoughts and feelings are not reality; they are the creations of my mind. So when there is an alarm, I am aware of my fears and my intrusive thoughts but they do not paralyze me. I can breathe and make myself relax; then I can be active and do everything I have to, run to the shelter and help my students or my own children at home. That is a very big positive change for me”.

Many of the teachers were very open and claimed that “the fear never goes away”, even with the help of mindfulness skills. However, the volume of the anxiety decreased and the focus on the here and now served as an anchor for active coping.

Finally, the teachers noted the impact of mindfulness training on their role as teachers as well as its influence on their students, in several important aspects of daily conduct in class.

All of the teachers emphasized that following their mindfulness training, their ability to take a step back and not react emotionally to infractions by their students, has become a major force in their professional conduct. Sivan said, “With the help of the training, I have learned to observe my students and their needs and to be more focused on helping them, especially when it comes to the more challenging ones”. Dikla stated, “There is one student in class. Time and again she has tested my boundaries, and at first, the whole class followed her. I told myself, take a deep breath, and be nice to her, although she deserves no such reaction. In the end, I was the one who triumphed!”.

Some of the teachers noted that they aimed to teach their students some of the principles and skills they had acquired during the program, thus implementing one of goals of the second year of training.

Yael explained, “There is this student who tends to act with no concern for the consequences. One day after class, I asked him to practice a breathing technique with me that I had learnt at the training program. He refused at first, but finally agreed. Nowadays, I see him take a breather and relax, before acting out”. Shosh pointed out, “We speak the language of mindfulness in class, emphasizing the need to be attuned to the needs of each student, to stretch a helping hand to those struggling. From a class with raging and arguing students, they have become a calm and cohesive group”. Another aspect noted was acceptance of members of minority groups by the students. Aliza said: “I have an assistant teacher, Nadav, who recently came out of the closet. Prior to my training, the kids used homosexual as an insult. Following the training, I gathered the children with Nadav present, and explained that there was nothing wrong with homosexuals and emphasized the need to accept one another’s lifestyle. The kids now refrain from calling each other homosexuals.” Another example was described by Aviva: “An Ethiopian student joined my class. The school headmaster explained to me that she believed that my pupils, following their familiarity with mindfulness, would accept that student more easily than any other class. She was right, as the other students accepted the Ethiopian girl warmly and helped her integrate quickly”.

The last aspect discussed was an improved ability to help students deal with the stressful security situation, yet noting the need to receive more professional guidance. Naama maintained, “In the second year of the training, I taught them [the students] some breathing skills and we spoke a lot about here and now and on focusing. However, I am not a professional mindfulness guide; there is very little that I can do. To my mind, incorporating mindfulness within the school curriculum, would benefit both students and staff”. The teachers stressed the contribution of the long training, which enabled them to experience mindfulness personally as well as pass it on to their students in some ways; nevertheless they felt that they were not professional enough to teach it actively as part of their class curriculum.

In summary, all the teachers suggested that long term mindfulness training could help teachers who live in conflict zones cope with stressful situations, both personally and professionally, and enhance their coping skills and their self-compassion.

## 4. Discussion

Coping with terror by civilians, especially in the Israeli context, is a subject of ongoing interest to scholars [1,22] who assess problem-focused and emotion-focused coping [3] in the context of living under the threat of terror. The current effort aimed to add to this literature by looking into the effects of mindfulness training, considered to have positive effects among teachers [16,21], and especially among Israeli female teachers living in a conflict zone. In general, the current results corroborate previous suggestion that mindfulness training enhances its practitioners’ ability to assess both the stressors they face and their available resources to handle these stressors in a more serene manner [6]. This means that mindfulness training helps to improve the employment of primary and secondary appraisals.

Following their participation in the training, several changes were noted by the interviewees. First, they indicated that mindfulness training helped them to regulate their fear reactions when under the threat of terror, both at home and at school. In this respect, employment of problem focused coping, instead of emotion focused coping, was attributed to participation in the program. Second, when conducting their relations with their family members, again, problem focused coping was the dominant mode of conduct noted, following the program. This was also the case at school, along with being able to express more compassion toward their students as well as their peers. This finding echoes those previously discussed noting the positive impact of mindfulness training on these aspects of conduct [14,15].

This is most likely the result of a change related to self-compassion following the program. It was argued that self-compassion indicates the awareness that each one is struggling to achieve, and thus acting with kindness to oneself leads to acting kindly to others, thus becoming more compassionate toward them [17]. Similar to the reports in the literature [19], the teachers pointed to a more positive classroom climate and management. It is worth noting that the teachers pointed out that the tools they have acquired to help them deal personally with stressful situations should be expanded further in order to assist them in teaching mindfulness to students. In this respect, it seems that these teachers acknowledge that the current training they have received has it limits, and they need further instruction on how to implement the guidelines offered by mindfulness training within their classes.

This study is the first of its kind, as it aimed to assess the impact of long-term mindfulness training among teachers living and working in an area, time and again struck by terror, noting its positive impact both at the professional and family levels. These findings suggest long-term mindfulness training should be part of the professional tool kit available to teachers living and working in areas dealing with the dangers of ongoing terror, and potentially also be accessible to their students.

However, several research limitations require future studies in order to expand these initial findings. First, coping was assessed through self-report only, with no control group comparison. Thus, in order to further explore the insights participants discussed, such an evaluation is needed. Second, participants had long-standing careers as teachers, and as such, their motivation was to acquire tools to manage the stressful situations they experienced as teachers in a conflict zone. This directs attention to the need to assess how novice teachers in conflict zones, who must also deal with the stressors emerging from their new career, discuss the impact of their participation in mindfulness training. A gender difference should also be researched, looking at the contribution of mindfulness training to males in comparison to females. Finally, a comparison between short- and long-term mindfulness training programs in Israel as well as in other areas where teachers must handle the threat of terror and war as part of their work should be examined.

## 5. Conclusions

The emerging picture that the current study suggests is that female teachers working and living in a conflict zone do benefit from long-term mindfulness training in the professional domain as well as when it comes to their individual and family conduct. This implies that in order to understand more fully the impacts of such training, attention should also be directed at populations of teachers in societies under the conditions of ongoing conflict in other parts of the world as well as to post-conflict societies. It is also important to find out how the students of teachers, like those studied here, reflect upon the outcomes following the conduct of their teachers as well as their family members.
